# An sRNA Screen for Reversal of Quinolone Resistance in *Escherichia coli*

**DOI:** 10.1534/g3.119.400199

**Published:** 2019-11-19

**Authors:** Kamya Bhatnagar, Aaron Hinz, Melissa Kohlman, Alex Wong

**Affiliations:** Department of Biology, Carleton University, Ottawa, ON, Canada K1S 5B6

**Keywords:** Antimicrobial resistance, *Escherichia coli*, fluoroquinolone, bacterial small RNA, drug targets, adjuvant

## Abstract

In light of the rising prevalence of antimicrobial resistance (AMR) and the slow pace of new antimicrobial development, there has been increasing interest in the development of adjuvants that improve or restore the effectiveness of existing drugs. Here, we use a novel small RNA (sRNA) screening approach to identify genes whose knockdown increases ciprofloxacin (CIP) sensitivity in a resistant strain of *Escherichia coli*. 5000 sRNA constructs were initially screened on a *gyrA* S83L background, ultimately leading to 30 validated genes whose disruption reduces CIP resistance. This set includes genes involved in DNA replication, repair, recombination, efflux, and other regulatory systems. Our findings increase understanding of the functional interactions of DNA Gyrase, and may aid in the development of new therapeutic approaches for combating AMR.

The rapid evolution of antimicrobial resistance (AMR) among bacterial pathogens and the slow development of new antibiotics have driven the search for novel approaches to counteract the resistance crisis (Levy and Marshall 2004; Davies and Davies 2010; Spellberg *et al.* 2004; Payne *et al.* 2007). Recently, re-sensitization of resistant strains to existing antibiotics has emerged as a promising strategy (Wright 2000; Bassetti *et al.* 2008). Compounds that reduce bacterial resistance, and that thereby restore the effectiveness of existing drugs, are a promising variety of antibiotic adjuvant (Wright 2016; González-Bello 2017). Certain β-lactamase inhibitors, for example, restore susceptibility to cephalosporins by inhibiting degradative enzymes (extended-spectrum β-lactamases, ESBLs) that are often responsible for resistance (Drawz and Bonomo 2010; King *et al.* 2014).

Here, we use a bacterial small RNA (sRNA) screen to identify genes whose knockdown re-sensitizes DNA gyrase-mediated resistance to the fluoroquinolone antibiotic ciprofloxacin (CIP). CIP is a synthetic antibiotic used globally for the treatment of many bacterial infections (Hooper 1999; Hooper and Rubinstein 2004; Bolon 2011); high-level resistance is typically conferred by mutations in the *gyrA* gene, which encodes one subunit of DNA gyrase, the primary target of quinolones (Drlica and Zhao 1997; Walsh 2000). The S83L substitution in the GyrA subunit confers a high level of CIP resistance in *E. coli* (Bagel *et al.* 1999; Bhatnagar and Wong 2019). We reasoned that CIP susceptibility might be restored in *gyrA* mutants by disrupting genes involved in the function of DNA Gyrase, or by altering cell permeability to CIP.

Bacterial sRNAs are widespread, non-coding RNA molecules. They are typically 50-300 nucleotides in size, *trans*-encoded, with distinct stem loops as secondary structures (Argaman *et al.* 2001; Gottesman 2004; Vogel and Sharma 2005, Sharma and Vogel 2009; Yoo *et al.* 2013). They play prominent roles in bacterial physiology by controlling gene expression post-transcriptionally. Each sRNA consists of two important regions. One is the recognition region that regulates sRNA-mRNA base-pairing through antisense short complementary base-pairing with the 5′ untranslated region (UTR) or translation initiation region (TIR), and the other is the scaffold (Hfq) region that stabilizes sRNA-mRNA base-pairing (Møller *et al.* 2002; Zhang *et al.* 2002; Storz *et al.* 2004; Jousselin *et al.* 2009; Vogel and Luisi 2011; Holmqvist and Vogel 2013; Vazquez-Anderson and Contreras 2013; Lee and Moon 2018; Lee *et al.* 2019). Binding of sRNA to mRNA targets can reduce gene expression by inhibiting translation or promoting mRNA degradation.

In eukaryotes, RNA interference (RNAi) is used extensively for studies of gene function. RNAi mediated gene silencing through short interfering (siRNA) and short hairpin (shRNA) RNAs has become a mainstay in cancer research and is a recognized basis of target validation and drug development (Silva *et al.* 2005, 2008; Schlabach *et al.* 2008; Scholl *et al.* 2009). In prokaryotes, comparable use of sRNA as a genetic tool is promising: proof-of-principle studies have demonstrated sRNA-mediated knockdown of protein levels, and several successful screens have been carried out (Nakashima *et al.* 2006; Meng *et al.* 2012; Man *et al.* 2011; Sharma *et al.* 2011, 2013). In the context of AMR, Lee *et al.* (2011) used a targeted sRNA screen to identify 45 genes whose knockdown reduced resistance to at least one of seventeen clinically relevant antibiotics, including essential genes that would be missed in knockout-driven screening approaches.

In this study, we randomized the antisense sequences of three naturally occurring sRNAs to generate an sRNA expression library with the potential to target diverse mRNA transcripts. We identified a number of sRNA sequences that reduce quinolone resistance on a *gyrA* S83L background. Further bioinformatic and functional analyses confirmed several genes whose down-regulation reduces resistance levels, and that may thus be promising adjuvant targets.

## Materials and Methods

### Bacterial strains, media and plasmid construction

One shot Top10 *E. coli* (Invitrogen, F- mcrA Δ(mrr-hsdRMS-mcrBC), j80lacZΔM15, ΔlacX74 recA1 araD139 Δ(ara leu)7697, galU, galK, rpsL (StrR), endA1, nupG) chemically competent cells were used for the development of randomization methods and for vector maintenance. For the sRNA screen, a quinolone resistant derivative of *E. coli* K-12 (MG1655) was generated by gene gorging (Herring 2003). Briefly, a fragment of *gyrA* encoding the S83L substitution (via a TCG->TTG mutation) and an I-SceI restriction site was generated by megaprimer PCR (Herring 2003) and cloned into the PCR2.1 vector using TOPO cloning (Invitrogen). This donor plasmid was co-transformed into *E. coli* K-12 (MG1655) along with the mutagenesis plasmid pACBSR. I-SceI endonuclease and λ-red functions encoded on pACBSR were then induced with arabinose. Potential mutants were plated on LB, and replica plated to LB+50 μg/ml kanamycin, LB+25 μg/ml chloramphenicol, or LB+25ng/ml ciprofloxacin to identify clones that had incorporated the S83L substitution and lost the donor and mutagenesis plasmids. Successful mutagenesis was confirmed by Sanger sequencing.

Cultures were grown in Lysogeny broth/agar cultures (LB) (10 g/l tryptone, 5 g/l yeast extract, 10 g/l NaCl; Bishop) at 37° throughout this study. LB media supplemented with 100μg/ml ampicillin (Sigma-Aldrich) was used for plasmid maintenance. Susceptibility assays were performed using CIP (Sigma-Aldrich) at 100ng/ml.

### Randomized library construction

**Figure 1:**
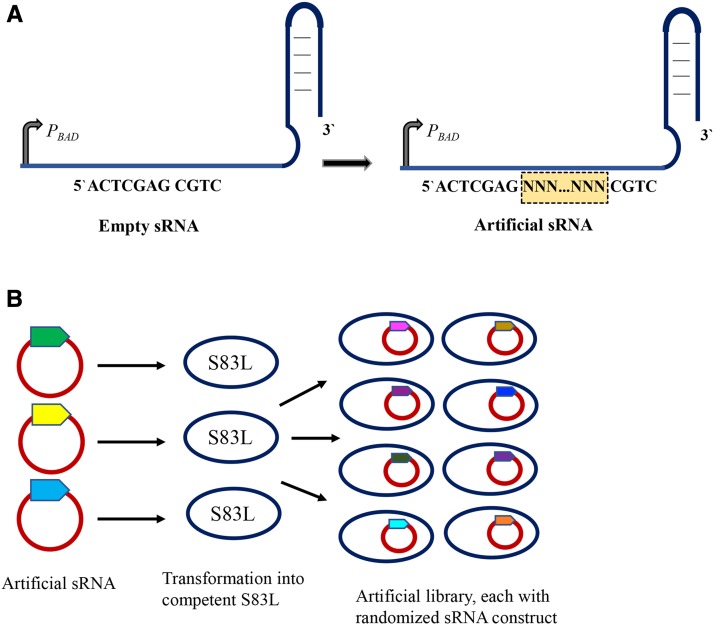
A schematic representation for the construction of the randomized artificial sRNA library. (A) The antisense domain is randomized with ∼10-30 randomized bases. The randomized bases were incorporated upstream of the scaffold regions of DsrA, MicF and Spot42 by PCR amplification of source vectors lacking the antisense regions (“empty” sRNA). (B) The subsequent randomized product was transformed into a *gyrA* S83L mutant to obtain a randomized artificial library, with each clone bearing a novel sRNA construct.

The small RNA expression vectors pBad- DsrA, MicF and Spot42 (Sharma *et al.* 2011; kindly donated by Yohei Yokobayashi, Okinawa Institute of Science and Technology) were used as templates for polymerase chain reaction (PCR) reactions. Randomized artificial sRNAs were constructed by incorporating random sequences in the antisense domains of these sRNAs using PCR primers with degenerate bases (Figure 1A). The native antisense domains of the sRNA scaffolds were absent from these constructs. Platinum Pfx polymerase (Invitrogen) was used to PCR amplify the whole plasmid with a common reverse primer Sartrev containing 20 degenerate bases and an sRNA specific forward primer consisting of 10 degenerate bases for randomizing the antisense domain. The complete list of oligonucleotides used in this study is provided in Supplemental Material, Table S1.

The PCR amplicon was purified (Bio Basic) and digested using the *Dpn*I enzyme (New England Biolabs) for 2 hr at 37° to cleave the methylated template parental DNA. Digested plasmids were phosphorylated using T4 polynucleotide kinase (New England Biolabs) for 20 min at 37°. After phosphorylation, the linear DNA was self-ligated using T4 DNA ligase (New England Biolabs) and incubated at 25° overnight.

The consistency of the randomization protocol was first verified in Top10 cells. Subsequently, the randomized sRNA library was introduced into an *E. coli gyrA* S83L mutant by transforming chemically competent cells with the self-ligated PCR amplicons. Transformations were plated on LB agar supplemented with ampicillin, single colonies were picked, grown overnight at 37° at 150 rpm with 100μg/ml ampicillin, and glycerol stocks were prepared in 96 well plates for further screening. Controls in each of the 96 well plates included *gyrA* S83L carrying empty sRNA plasmid and untransformed *E. coli* MG1655. Libraries were constructed separately for each of the three expression vectors, consisting of ∼5000 transformants in total. ∼40 plasmids from the randomized library (roughly equal numbers from each plasmid backbone) were extracted and sent for Sanger sequencing to confirm randomization.

### Ciprofloxacin sensitivity screen

In order to identify sRNA constructs that increased CIP sensitivity, 24 h growth curves in CIP-containing media were assayed for ∼5000 *gyrA* S83L mutant clones bearing randomized constructs. Cultures were inoculated at a 1:100 dilution from glycerol stock cultures and grown overnight at 37°, 150 rpm in LB with 100μg/ml ampicillin for sRNA plasmid maintenance. For 24 h growth curves, the overnight cultures were diluted (1:100) in LB supplemented with 100ng/mL CIP, 0.5mM arabinose, and 100μg/ml ampicillin. Arabinose is required to induce expression of the sRNA construct. 100 ng/mL CIP was chosen as an intermediate concentration between the minimum inhibitory concentration (MIC) of the WT (30 ng/mL) and the S83L mutant (600 ng/mL). The OD_600_ of each culture was measured on a Biotek ELx808 plate reader every 37 min for 24 h, incubating at 37° with 30 s of shaking every 5 min. Two growth parameters, lag time and maximum growth rate, were estimated using the program GrowthRates (Hall *et al.* 2014). For each well, OD_600_ at time zero was used as a well-specific blank.

Clones showing growth repression in the primary CIP sensitivity screen were selected for secondary screening in the presence and absence of CIP (100ng/ml) using similar experimental methods as described above. Clones whose growth was repressed in the presence of CIP, but not in LB alone, were selected for further analysis.

### Target identification

In order to identify potential target mRNAs, sRNA vectors were extracted from CIP-sensitive clones and their sRNA regions were Sanger sequenced. Putative mRNA targets were identified for the randomized sRNA sequences using the online software packages IntaRNA (Mann *et al.* 2017), Target RNA (Kery *et al.* 2014) and RNA predator (Eggenhofer *et al.* 2011). These web based programs predict hybridizations between two RNA molecules, and provide a graphical overview of the sRNA-mRNA binding interactions (Tjaden 2008; Smith *et al.* 2010; Eggenhofer *et al.* 2011). The resulting target genes were identified and selected for experimental validation.

### Validation for selected targets

Candidate genes whose knockdown may induce CIP sensitization were validated experimentally by constructing double mutants, in which the *gyrA* S83L mutation was combined with knockout mutations of the computationally predicted target genes. The knockout mutants were selected from the Keio mutant collection (Baba *et al.* 2006), wherein a kanamycin resistance cassette was used to replace non-essential genes. The *gyrA* S83L mutation was introduced by oligonucleotide-mediated recombineering (Ellis *et al.* 2001) into the Keio deletion mutants, to construct each double mutant. A mobilizable derivative of pMA7SacB was used (Lennen *et al.* 2015), in which an arabinose-inducible promoter (P_BAD_) controls expression of the β subunit of λ-Red recombinase and the *E. coli* Dam methylase. Dam methylase induction has been shown to increase mutagenesis efficiencies in *E. coli*, while reducing off-target effects, by transiently interfering with the DNA mismatch repair system (Lennen *et al.* 2015). The plasmid was introduced into recipient strains by conjugation using a donor *E. coli* strain (WM3064) that is auxotrophic to diaminopimelic acid (DAP) (Dehio and Meyer 1997; Saltikov and Newman 2003). Recipients (Keio mutants) were cultured in LB broth supplemented with 30 μg/ml kanamycin and the donor was cultured in LB supplemented with 0.3 mM DAP and 100 μg/ml ampicillin (for Red plasmid maintenance). Mating mixtures were spotted on LB agar with 0.3 mM DAP and incubated overnight. Exconjugates were selected with 100 μg/ml ampicillin on LB lacking DAP, to prevent growth of donors. λ-Red recombination was performed on Keio mutants harboring the Red plasmid as previously described (Lennen *et al.* 2015) using an oligonucleotide encoding the *gyrA* (S83L) mutation (5′-AACGCAGCGAGAATGGCTGCGCCATGCGGACGATCGTGTCATAGACCGCCAAGTCACCATGGGGATGGTATTTACCGATTACGTCACCAA-3′). A single round of recombination was carried out to minimize off-target effects. Transformants harboring the *gyrA* mutation were selected by plating on LB supplemented with 30 μg/ml ciprofloxacin and 30 μg/ml kanamycin. The strains were cured of the λ Red plasmid by *sacB* counterselection by streaking on LB agar containing 5% sucrose. The *gyrA* gene of each putative double mutant was PCR amplified and Sanger sequenced to verify the presence of the S83L mutation (PCR primers: Gyrase forward - 5′GTAAAACGACGGCCAGTGATGAGCGAC3′, Gyrase reverse - 5′CGGTACGGTAAGCTTCTTC3′).

### Ciprofloxacin susceptibility assay

Minimum inhibitory concentrations (MIC) for CIP were determined for each of the *gyrA* S83L/ Keio knockout double mutants using a 96 well plate assay. Antibiotic concentrations were started at 125ng/ml, 4ug/ml, and 8ug/ml for Keio single knockout mutants, double mutants (Keio single knock out mutant + *gyrA* S83L), and controls (*cybC* only and *cybC* + *gyrA* S83L) respectively, and were diluted in a twofold series and dispensed with 125μl/well of LB into 96-well plates. The 96 well plates were incubated overnight at 37°, with shaking at 150 rpm. The MIC was defined as the lowest concentration of antibiotic for which 90% growth inhibition was visibly observed after overnight culture.

### Data availability

Strains and plasmids are available upon request. File S1 contains the data presented in this manuscript. Supplemental material available at figshare: https://doi.org/10.25387/g3.7877543.

## Results

### Randomized sRNA library construction

We generated a library of artificial sRNAs by randomizing the antisense domain sequences of three native *E. coli* sRNAs: DsrA, MicF, and Spot42. We transformed the sRNA library into an *E. coli gyrA* S83L mutant and collected a total of ∼5000 clones, each bearing a plasmid with an artificial sRNA construct (Figure 1A, B). We arbitrarily isolated 40 clones from the plasmid library and sequenced their sRNA regions to check for the incorporation of randomized sequences. The sequencing results confirmed the presence of unique randomized sequences in each of the engineered sRNA constructs (example sequences are shown in Table 1).

**Table 1:** Randomized sequences of artificial sRNAs constructs. ∼10-30 randomized bases (bold) were incorporated upstream of the scaffold regions of DsrA, MicF and Spot42 by PCR amplification of source vectors lacking the antisense regions (“empty” vectors). All of the sRNAs start from the vector-derived sequence of 5`ACTCGAG (*italics*). Example randomized sequences are shown for each construct (2 or 3, as indicated in parentheses)

Scaffold	Randomized region
Empty DsrA sequence	*actcgag*caattttttaagtgcttcttgcttaag *actcgag***ttgtacctgctttcgatacgactttcat**caattttttaagtgcttcttgcttaag
Example randomized DsrA (3)	*actcgag***ttctctcgtcggactgaacgtggagctgg**aattttttaagtgcttcttgcttaag
	*actcgag***gaattcggaccatgataccactgagttt**aattttttaagtgcttcttgcttaag
Empty MicF sequence	*actcgag*cgtcattcatttctgaatgtctg *actcgag***tccccttcacgggtgcaacgggccaatgg**cgtcattcatttctgaatgtctg
Example randomized MicF (3)	*actcgag***ctatacagatcatttacaccccgtacatc**cgtcattcatttctgaatgtctg
	*actcgag***ggtagtagactg**cgtcattcatttctgaatgtctg
Empty Spot42 sequence	*actcgag*atttggctgaatattttagccgc *actcgag***ggaggggggg**atttggctgaatattttagccgc
Example randomized Spot42 (2)	*actcgag***ccgtatagaaccacatctgcctggggggg**atttggctgaatattttagccgc

### Ciprofloxacin sensitivity screening

**Figure 2:**
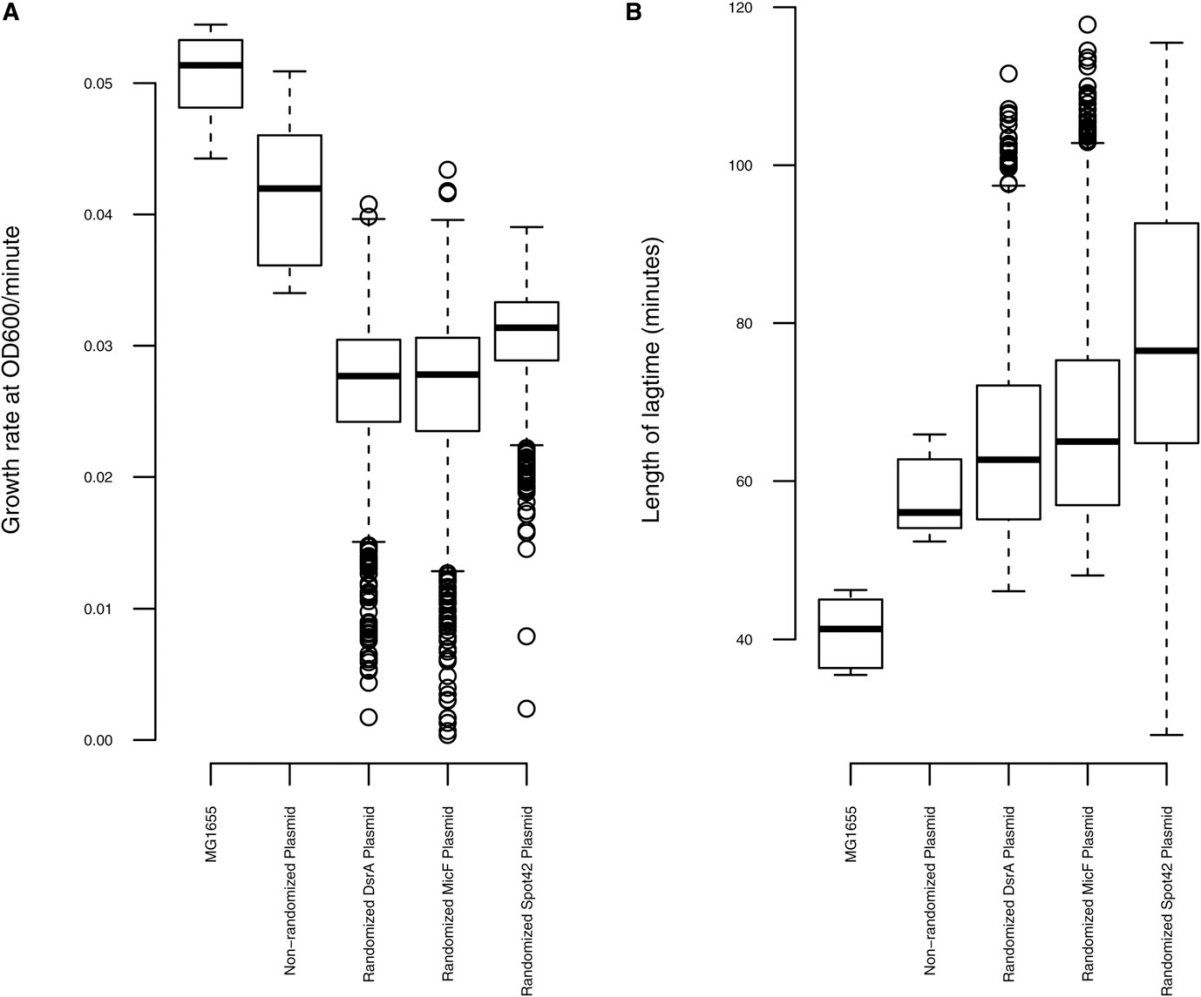
Boxplot distributions of CIP-sensitivity phenotypes for ∼5000 *gyrA* S83L mutants harboring randomized sRNA plasmid constructs. The growth rate (A) and lag time (B) distributions are depicted for clones cultured in LB with 600 ng/ml CIP. *E. coli* MG1655 grown without CIP, and *gyrA* S83L mutants bearing empty sRNA plasmids grown with CIP, were used as controls. The boxplots give the median and first and third quartiles, with whiskers showing either the maximum (minimum) value or 1.5 times the interquartile range of the data, whichever is smaller (larger). The outliers (528 clones) selected for further investigation have substantial reductions in growth rate and/or increased lag phase.

To identify clones with increased susceptibility toward CIP, all ∼5000 *gyrA* S83L clones bearing sRNA constructs were grown in LB supplemented with 100ng/mL CIP and arabinose to induce sRNA expression. The MIC of the *gyrA* S83L mutant is 600ng/ml, so we reasoned that incubation at this sub-MIC concentration of CIP would be a good indicator for changes in sensitivity. Controls included wild-type *E. coli* MG1655 grown in LB without CIP (median growth rate: 0.06 OD_600_ /minute and length of lag phase: 41 min), and a *gyrA* S83L mutant bearing a non-randomized plasmid grown in LB+CIP (median growth rate: 0.05 OD_600_ /minute and length of lag phase: 47.8 min) (Figure 2). We selected a total of 528 clones exhibiting substantial reductions in growth rate and/or increased lag phase (outliers from Figure 2). From the individual sRNA scaffolds, we selected 199 clones from DsrA (median growth rate: 0.04 OD_600_ /minute and length of lag phase: 62.1 min), 231 clones from MicF (median growth rate: 0.04 OD_600_/minute and length of lag phase: 64.6 min), and 98 clones from Spot42 (median growth rate: 0.05 OD_600_/minute and length of lag phase: 60 min).

**Figure 3:**
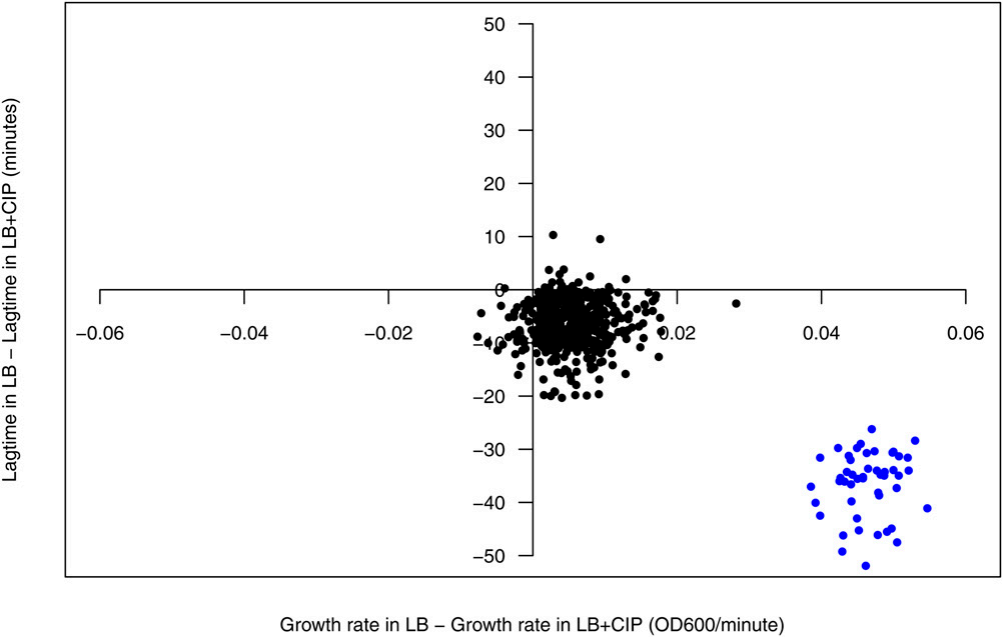
Effects of sRNA expression on growth rate (OD_600_/minute) and length of lag phase (minutes) with and without CIP. The 48 selected constructs showing repression in growth in the presence of CIP are indicated in blue.

We repeated the growth curves of the 528 putatively CIP susceptible clones in the presence and absence of CIP (Figure 3, Figure S1). This secondary screen was performed in order to verify the CIP-sensitive phenotypes and to determine whether or not the growth deficiencies were specific to CIP; a growth deficit in LB alone would indicate a general fitness effect of the sRNA, rather than reversal of CIP resistance. From this secondary screen, we selected 48 clones showing little or no growth inhibition in LB without CIP (Mean growth rate: 0.07 OD_600_/minute and length of lag phase: 48.1 min), but whose growth was inhibited in LB supplemented with CIP (Mean growth rate: 0.02 OD_600_/minute and length of lag phase: 85 min).

### Identification of target genes

We sequenced the randomized antisense regions of the sRNA plasmids isolated from the 48 clones exhibiting growth defects specific to the presence of CIP. Candidate target mRNAs were identified using the online tools IntaRNA, Target RNA and RNA predator (Mann *et al.* 2017; Kery *et al.* 2014; Eggenhofer *et al.* 2011). These packages predict target mRNA sites in the *E. coli* MG1655 transcriptome with the potential for complementary sRNA interactions. We detected complementary hits for 31/48 randomized sequences (an example of complementarity between an artificial sRNA and its predicted target gene (*sbmC*) is shown in Figure S2). Since these tools identify complementary matches between short sequences, several sequences were matched to multiple targets, resulting in a total of 222 potential gene targets (Table S2). Candidate targets included genes involved in DNA repair and recombination (*recC*, *recD*, *recJ*, *sbmC*), the SOS response and error prone replication (*umuC*, *xseA*), transcriptional regulation (*yeeY*, *mtlR*), cell wall division and assembly (*zipA*, *minC*), transport and efflux (*emrB*, *tolQ*), two-component regulatory systems (*cpxA*, *cpxR*, *ompR*, *cheB*), and chaperone proteins (*hybB*, *dnaK*).

### Experimental validation of targets

We carried out validation experiments for 36 genes that encoded potential mRNA targets (Table 2). Since CIP primarily interacts with DNA Gyrase, we focused on putative target genes related to Gyrase and its functions in DNA replication, repair and recombination. Additional genes were chosen because of their roles in efflux transport systems, two-component regulatory systems, transcriptional regulators and as chaperone proteins (Table 2). For each candidate target gene, the corresponding knockout mutant was obtained from the Keio single gene knockout collection (Baba *et al.* 2006). The *gyrA* S83L mutation was then transferred into the Keio clone by λ-Red mutagenesis, generating a double mutant.

**Table 2:** 36 genes selected for experimental validation, encoding predicted sRNA targets associated with reductions in *gyrA* S83L mediated CIP resistance. Target gene function, category, and locus tags were obtained from the EcoCyc database (Keseler *et al.* 2016) (http://www.ecocyc.org).

S.NO.	Target gene	Function	Category	Locus tag	Reference
1	*recC*	DNA helicase, ATP-dependent dsDNA/ssDNA exonuclease	DNA replication, repair and recombination	b2822	Persky and Lovett (2008)
2	*recD*	Exonuclease V (RecBCD complex) alpha chain	DNA replication, repair and recombination	b2819	Persky and Lovett (2008)
3	*recJ*	ssDNA exonuclease; 5′ 3′ specific	DNA replication, repair and recombination	b2892	Han *et al.* (2006)
4	*rnt*	Ribonuclease T (RNase T)	DNA replication, repair and recombination	b1652	Viswanathan *et al.* 1999)
5	*rpoN*	RNA polymerase sigma 54 (sigma N) factor	DNA replication, repair and recombination	b3202	Zhang *et al.* (2016)
6	*smrB*	Putative DNA endonuclease	DNA replication, repair and recombination	b2331	Keseler *et al.* (2016)
7	*sbmC*	DNA gyrase inhibitor	DNA replication, repair and recombination	b2009	Nakanishi *et al.* (1998)
8	*tus*	Inhibitor of replication at Ter; DNA binding protein	DNA replication, repair and recombination	b1610	Hill *et al.* 1989
9	*topB*	DNA topoisomerase III	DNA replication, repair and recombination	b1763	DiGate and Marians (1988)
10	*umuC*	DNA polymerase V; subunit C	DNA replication, repair and recombination	b1184	Tang *et al.* (1999)
11	*uvrD*	DNA-dependent ATPase I and helicase II	DNA replication, repair and recombination	b3813	Matson and Robertson (2006)
12	*xseA*	Exonuclease VII large subunit	DNA replication, repair and recombination	b2509	Vales *et al.* (1982)
13	*dnaQ*	DNA polymerase III epsilon subunit	DNA replication, repair and recombination	b0215	Echols *et al.* (1983)
14	*helD*	DNA helicase IV	DNA replication, repair and recombination	b0962	Mendonca *et al.* (1993)
15	*priB*	Primosomal protein N	DNA replication, repair and recombination	b4201	Heller and Marians (2005)
16	*priC*	Primosomal replication protein N	DNA replication, repair and recombination	b0467	Heller and Marians (2005)
17	*rnr*	Exoribonuclease R; RNase R	DNA replication, repair and recombination	b4179	Cheng and Deutscher (2002)
18	*uvrA*	ATPase and DNA damage recognition protein of nucleotide excision repair	DNA replication, repair and recombination	b4058	Wagner *et al.* (2009)
19	*yeeY*	Predicted DNA binding transcriptional regulator	Transcriptional regulators	b2015	Keseler *et al.* (2016)
20	*mtlR*	Transcriptional repressor (mannitol)	Transcriptional regulators	b3601	Figge *et al.* (1994)
21	*ydaG*	Uncharacterized protein	Uncharacterized protein	b1355	Keseler *et al.* (2016)
22	*ydaV*	Uncharacterized protein (predicted ATP binding protein)	Uncharacterized protein	b1360	Hidese *et al.* (2014)
23	*ompR*	Response regulator with EnvZ	Two component regulatory system	b3405	Cai and Inouye (2002)
24	*cheB*	Fused chemotaxis regulator protein	Two component regulatory system	b1883	Baker *et al.* (2006)
25	*cpxA*	Sensory histidine kinase with CpxR	Two component regulatory system	b3911	Bury-Moné *et al.* (2009)
26	*cpxR*	Response regulator with CpxA	Two component regulatory system	b3912	Batchelor *et al.* (2005)
27	*dnaK*	Chaperone Hsp70; co-chaperone with DnaJ	Chaperone protein	b0014	Mayer *et al.* (2000)
28	*hybB*	Predicted hydrogenase 2 cytochrome b type component	Chaperone protein	b2995	Menon *et al.* (1994)
29	*emrB*	Multidrug efflux system protein	Transport, Efflux system	b2686	Lomovskaya *et al.* (1995)
30	*tolA*	Membrane anchored protein in TolA-TolQ-TolR complex	Transport, Efflux system	b0739	Bernadac *et al.* (1998)
31	*tolQ*	Membrane spanning protein in TolA-TolQ-TolR complex	Transport, Efflux system	b0737	Bernadac *et al.* (1998)
32	*tolC*	AcrAB-TolC multidrug efflux transport system	Transport, Efflux system	b3035	Bernadac *et al.* (1998)
33	*minC*	Septum site determining protein, inhibitor of FtsZ ring polymerization	Cell division	b1176	Pichoff, and Lutkenhaus (2001)
34	*zipA*	FtsZ stabilizer	Cell division	b2412	Hale and de Boer (2002)
35	*proC*	Pyrroline-5-carboxylate reductase NAD(P)-binding	Catalytic, biosynthetic pathways	b0386	Deutch *et al.* (1982)
36	*pyrB*	Catalytic subunit	Catalytic, biosynthetic pathways	b4245	Donahue and Turnbough (1994)

**Figure 4:**
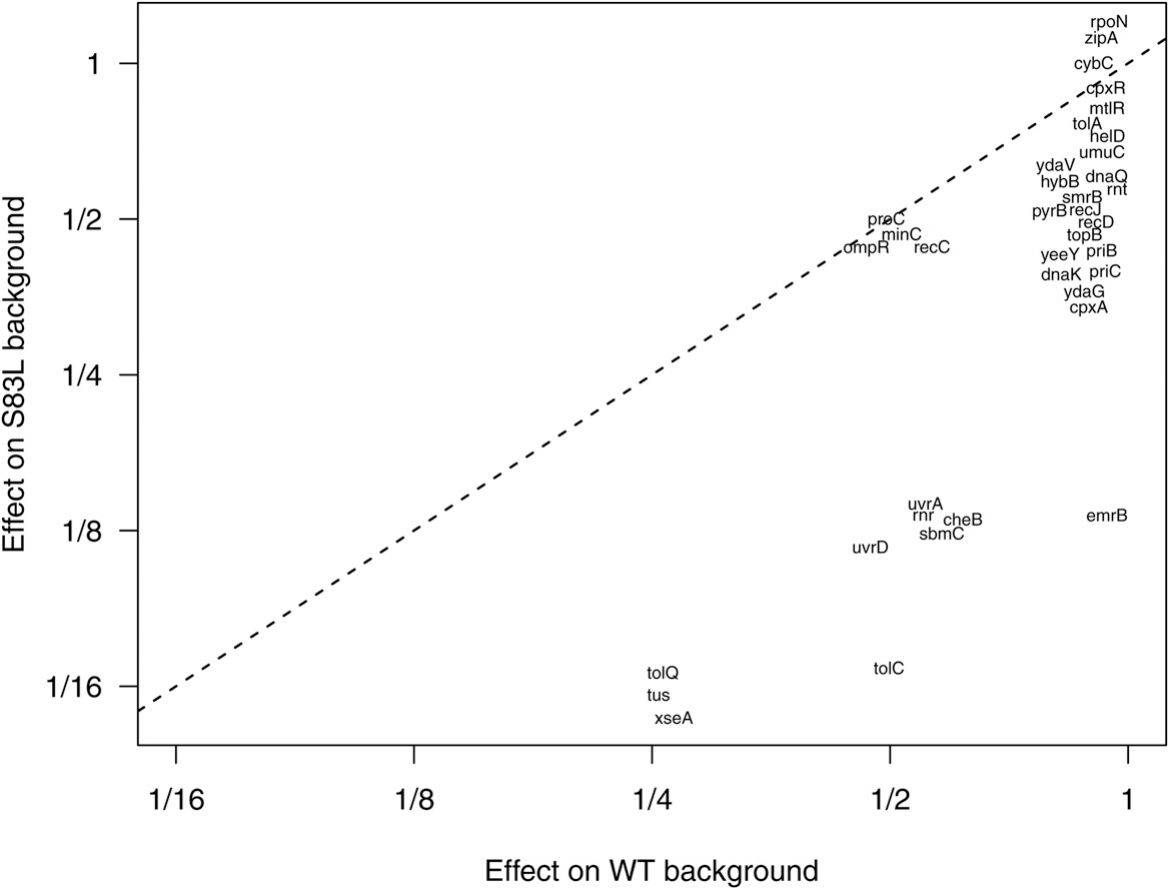
Fold change in MIC values for 36 knockout mutants on wild-type and *gyrA* S83L backgrounds. Absolute 1xMIC values for the S83L mutant and WT are 1000 ng/mL and 30 ng/mL, respectively. The 30/36 mutants that fall below the 1:1 line show reductions in CIP resistance on the *gyrA* S83L background but not on the wild-type background.

To determine which candidate target genes conferred increased sensitivity to CIP on the *gyrA* S83L background, we obtained the MIC of each double mutant and the corresponding single mutants (Table S3). Knockout of a pseudogene, *cybC*, was used as a control for the effect of the kanamycin cassette present in the Keio clones. The MIC values of a number of double mutants showed reduced susceptibility when compared to the *cybC*+S83L (MIC = 1000ng/ml) double mutant. Importantly, for 30 genes, the reduction in CIP resistance was more pronounced on the *gyrA* S83L background than on the WT background; these genes fall below the 1:1 line on Figure 4. Inactivation of these genes thus leads to substantial reductions in *gyrA*-mediated CIP resistance. While we did not explicitly account for off-target effects of the λ-Red recombineering used to generate the double mutants, off-target mutations are unlikely to account for the majority of reductions in MIC, since every double mutant was generated independently. Mutants showing a reduction in CIP MIC on the *gyrA* S83L background include *xseA*, *tus* (double mutant MIC reduction of 16-fold), *sbmC* (double mutant MIC reduction of eightfold), *tolQ* and *tolC* (double mutant MIC reduction of 16-fold).

## Discussion

We carried out an sRNA screen to identify genes whose knockdown restores quinolone sensitivity in *E. coli*. Following an initial screen of ∼5000 sRNA-bearing clones, and secondary screening of over 500 clones, we found 30 genes whose disruption increases sensitivity of a *gyrA* S83L mutant by twofold or more. Our findings expand knowledge of the genetic interaction network of the essential gene *gyrA*, and provide potential targets for the development of antibiotic adjuvants to restore sensitivity in quinolone resistant pathogens.

Chemical-genetic sensitivity screens have largely used knockout approaches, whereby a library of knockout mutants is screened for sensitivity or resistance to an antibiotic at sub-lethal concentrations (*e.g.*, Tamae *et al.* 2008; Breidenstein *et al.* 2008; Fajardo *et al.* 2008; Gomez and Neyfakh 2006; Liu *et al.* 2010). Typically, such screens are carried out on a wild-type, antibiotic susceptible background, so they are not well-suited to identifying genes whose knockdown reverses resistance. Nonetheless, Tamae *et al.* (2008) did show that knockouts of five genes recovered from a sensitivity screen (*recC*, *recA*, *fis*, *xseA*, *tolC*; all but *fis* were also found here) did reduce CIP resistance on a *gyrA* mutant background. sRNA screens offer a powerful means for identifying genetic-background specific effects, since sRNA libraries can be readily generated in any transformable strain (Sharma *et al.* 2011, 2013; Lee *et al.* 2011). Moreover, inducible sRNA constructs can be used for studying gene functions of essential genes as well as non-essential genes (Rodrigo *et al.* 2012), which is not possible with knockouts. The randomization approach that we have adopted here (see also Sharma *et al.* 2011, 2013) is particularly promising, since it does not require targeted cloning of gene-specific sRNAs. We note a need for additional efforts to test and refine sRNA scaffolds – it is unknown, for example, whether different scaffolds differ in the extent of knockdown achieved for their targets.

It is likely that a larger pool of sRNA clones would be needed to saturate the genome. It is not straightforward to calculate how many clones would need to be sampled, since each sRNA may target more than one gene. In the limiting case where each sRNA targets only a single gene, approximately 13600 sRNA clones would be needed to “hit” 95% of the 4539 genes in the *E. coli* genome (calculated as 1-exp(-13600/4539); gene number from ecocyc.org). If each sRNA knocks down more than one target RNA, however, fewer clones would be needed.

Direct use of sRNAs as therapeutics would require the efficient expression of a synthetic sRNA construct inside the target bacterial cell. This would require a competent delivery system, such as phage or a conjugative plasmid system. An engineered-phage approach illustrated by Lu and Collins (2009) expressed the LexA and OmpF proteins from phage M13. This resulted in reduction in antibiotic resistance evolution in mice when injected with engineered-phages. Thus, a comparable approach could be a plausible delivery system for combating resistant bacteria. An alternative strategy would be to identify small molecules that inhibit the targets identified here. For example, we showed that inhibition of *sbmC* reduces CIP resistance by twofold, and SbmC is a known target of the peptide antibiotic Microcin B17 (Baquero *et al.* 1995; Collin *et al.* 2011). Thus, sRNA-mediated target discovery may be an efficient option for generating prospects for novel therapeutics.
